# FOXO3a promotes gastric cancer cell migration and invasion through the induction of cathepsin L

**DOI:** 10.18632/oncotarget.8977

**Published:** 2016-04-25

**Authors:** Shan Yu, Yiyi Yu, Wen Zhang, Wei Yuan, Naiqing Zhao, Qian Li, Yuehong Cui, Yan Wang, Wei Li, Yihong Sun, Tianshu Liu

**Affiliations:** ^1^ Department of Medical Oncology, Zhongshan Hospital, Fudan University, Shanghai, People's Republic of China; ^2^ Department of Pathology, Zhongshan Hospital, Fudan University, Shanghai, People's Republic of China; ^3^ Department of Biostatistics, Fudan University, Shanghai, People's Republic of China; ^4^ Department of General Surgery, Zhongshan Hospital, Fudan University, Shanghai, People's Republic of China

**Keywords:** FOXO3a, cathepsin L, gastric cancer migration, invasion

## Abstract

Forkhead box O3A (FOXO3a) is an important transcription factor involved in various human cancers. However, the role of FOXO3a in regulating the invasion and metastasis of gastric cancer cells has not been clarified. Here, we report that FOXO3a overexpression promoted migration and invasion of gastric cancer cells by upregulating cathepsin L. FOXO3a knockdown suppressed migration and invasion and also downregulated cathepsin L expression in gastric cancer cells. Silencing cathepsin L in these cells suppressed FOXO3a overexpression-induced cell migration and invasion. Mechanistic studies revealed that FOXO3a increased cathepsin L promoter activation, and cathepsin L overexpression repressed E-cadherin expression, causing gastric cancer cells to undergo epithelial-mesenchymal transition (EMT). Our data reveal a previously unexplored function of FOXO3a in gastric cancer invasion by regulating proteins involved in extracellular matrix (ECM) degradation and EMT. We suggest that FOXO3a may be of prognostic value and a potential therapeutic target in blocking tumor metastasis.

## INTRODUCTION

Gastric cancer is one of the primary causes of death worldwide. Surgical resection combined with adjuvant therapy is efficacious in the early stages of the disease; however, subsequent relapse and metastasis are unfortunately common. When patients seek treatment at advanced stages, often little can be done, and prognosis is extremely poor, with an overall 5-year survival rate of approximately 15% [1, 2]. Therefore, it is crucial to elucidate the sophisticated molecular mechanisms underlying gastric cancer invasion and eventually metastasis to identify new predictive biomarkers and molecular targets for treatment.

The phosphoinositide 3-kinase (PI3K)/protein kinase B (PKB, also called AKT) signaling pathway has been shown to participate in the progression of gastric cancer. The forkhead/winged helix box class O (FOXO) transcription factors are downstream targets of the PI3K/PKB pathway. In mammals, the FOXO family contains four members (FOXO1, FOXO3a, FOXO4 and FOXO6), which activate or repress multiple genes involved in a wide spectrum of cellular functions, including cell proliferation, apoptosis, differentiation and resistance to reactive oxygen species and DNA damage [3–13]. In human cancers, FOXO3a was initially categorized as a tumor suppressor because inhibition of FOXO3a transcription promoted cell transformation, tumor progression, and angiogenesis [14–17], and our previous study also showed that activation of FOXO3a was correlated with good prognosis in human gastric cancer [18]. However, accumulating evidence has suggested that FOXO3a may also promote the survival and metastasis of cancer cells. It has been reported that FOXO3a facilitates tumor cell survival through the activation of NF-κB under stress conditions [19]. A recent publication showed that FOXO3a accelerates metastasis of colon cancer independently of the corresponding upstream pathways [20]. In doxorubicin-sensitive leukemic cells, the activation of FOXO3a by doxorubicin induced the expression of the multidrug resistance gene ABCB1 (MDR1), resulting in doxorubicin resistance [21]. Therefore, the function of FOXO3a should be clearly demonstrated in any specific cancer before it is considered to be an effective target of anti-cancer therapy [22–23]. However, there have been few detailed functional analyses of FOXO3a in gastric cancer.

In our previous work, we conducted a tissue microarray analysis and found that FOXO3a expression was significantly increased in gastric cancer tissues [18]. Next, we conducted functional assays using stable FOXO3a-transfected gastric cancer cell lines. FOXO3a overexpression promoted migration and invasion of gastric cancer cells, whereas FOXO3a knockdown suppressed migration and invasion in gastric cancer cells.

On the basis of the microarray and functional analyses, we hypothesized that FOXO3a may be a potential tumor promoter in gastric cancer. We then attempted to identify potential target genes of FOXO3a relating to invasion and migration. Cathepsin L, a cysteine protease, is produced as preprocathepsin L, transported by the Golgi apparatus in the form of procathepsin L, and eventually stored in lysosomes as mature cathepsin L. In addition to acting as regulator of the cell cycle [24] and immune system [25], cathepsin L also plays an important role in tumor migration and invasion. Once activated extracellularly, cathepsin L not only degrades the extracellular matrix but also reduces cell-cell adhesion through cleavage of E-cadherin [26]. We initially conducted a tissue microarray analysis and found that the expressions of FOXO3a and cathepsin L were positive correlated. We then used luciferase assays and western blot analysis to identify cathepsin L as a target gene of FOXO3a. Finally, we knocked down cathepsin L using an shRNA to confirm the mechanism of FOXO3a's tumor-promoting function.

## RESULTS

### Knockdown and overexpression of FOXO3a in gastric cancer cell lines

**Figure 1:**
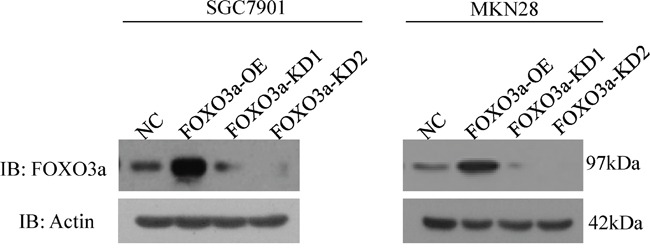
Knockdown and overexpression of FOXO3a in gastric cancer cells Western blot analysis of FOXO3a expression in SGC7901 and MKN 28 cells with FOXO3a knockdown (KD), overexpression (OE) or negative control (NC).

To investigate the functional role of FOXO3a in gastric cancer, FOXO3a-specific knockdown and overexpression constructs were stably expressed in two cancer cell lines, SGC7901 and MKN28 (Figure 1). Western blot results showed significantly reduced FOXO3a expression in the knockdown (KD) cell lines and highly expressed FOXO3a in the overexpression (OE) cells compared with the respective controls.

### Altered FOXO3a expression affects gastric cancer cell migration and invasion

**Figure 2:**
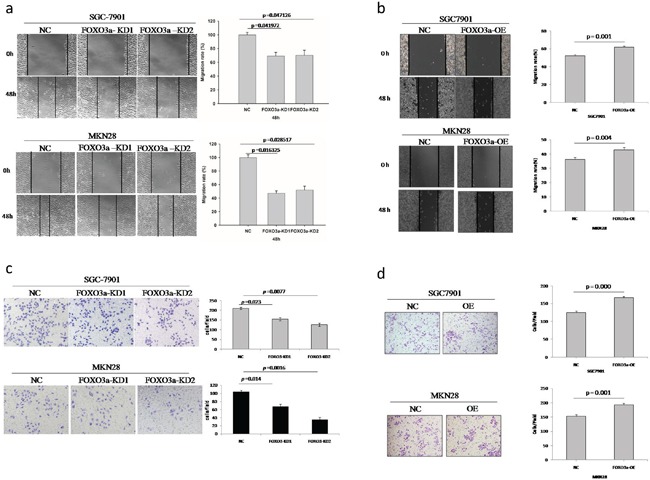
Migration and invasion assay of FOXO3a knockdown or overexpressing gastric cancer cells **a.** Depletion of FOXO3a in SGC7901 and MKN28 cells resulted in a significant decrease in wound healing. **b.** Over-expression of FOXO3a in SGC7901 and MKN28 cells resulted in a significant increase in would healing. **c.** Depletion of FOXO3a in SGC7901 and MKN28 cells resulted in a significant decrease in invasive migration. **d.** Over-expression of FOXO3a in SGC7901 and MKN28 cells resulted in significant increase in invasive migration. Images are representative of three independent experiments. Values are mean ± s.e.m.

To examine the effects of FOXO3a on the migration of two gastric cancer cell lines (SGC7901, MKN28), we conducted wound-healing assays. As shown in Figure 2, depletion of FOXO3a in these cells resulted in a significant decrease in wound healing after 48 hours, whereas overexpression of FOXO3a in these cells resulted in a significant increase in wound healing.

To further understand the role of FOXO3a in tumor invasion and metastasis, we analyzed two cell lines in standard Matrigel chemoinvasion assays. We assessed the ability of SGC7901 cells and MKN28 cells to invade Matrigel in a FOXO3a-dependent manner. As shown in Figure 2, depletion of FOXO3a in these cells resulted in a significant decrease in invasive migration, whereas overexpression of FOXO3a in these cells resulted in a significant increase in invasive migration.

However, FOXO3a had no effect on cell proliferation in both SGC7901 and MKN28 cells (Supplementary Figure 1).

### FOXO3a induces the expression of cathepsin L in gastric cancer cells

Cathepsin L plays an important role in degrading the ECM and promoting tumor invasion. To determine whether FOXO3a regulates the expression of cathepsin L, we analyzed cathepsin L expression by western blot analysis using SGC7901 and MKN28 cells.

**Figure 3:**
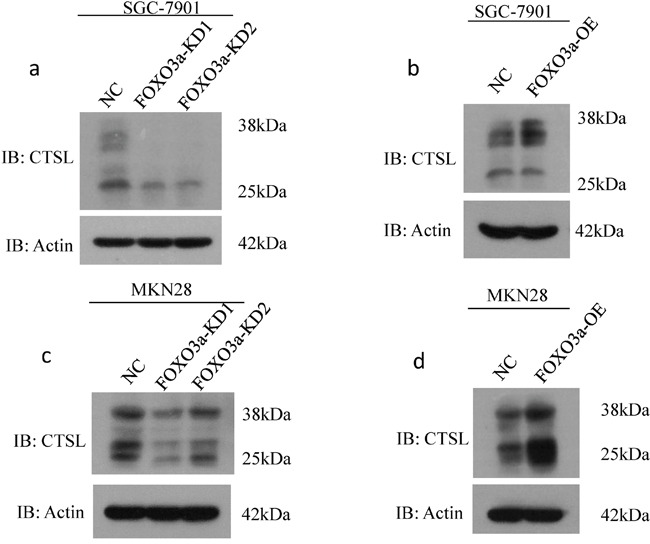
Western blot assay of cathepsin L expression in FOXO3a knockdown or overexpressing gastric cancer cells Western blot assay of cathepsin L (CTSL) expression in SGC7901 **a-b.** and MKN28 cells **c-d.** with FOXO3a knockdown and overexpressing, probed with antibody to β-actin.

As shown in Figure 3, we found that FOXO3a-depleted cells exhibited decreased expression of mature cathepsin L. By contrast, cells overexpressing FOXO3a had increased expression of mature cathepsin L, indicating that cathepsin L may be a direct transcriptional target of FOXO3a.

### Cathepsin L is coexpressed with FOXO3a in gastric cancer tissues

**Figure 4:**
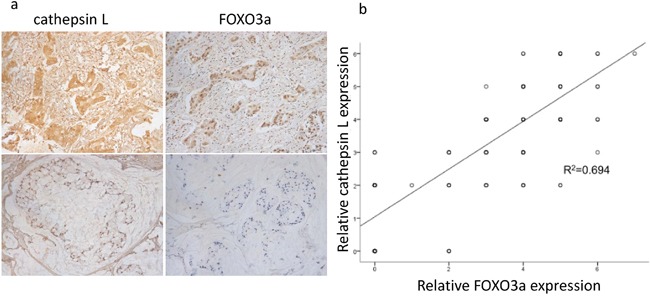
Positive correlation between FOXO3a and cathepsin L expression **a.** IHC expression of FOXO3a and cathepsin L in gastric cancer tissues. Upper: immunostaining of cathepsin L was positive in gastric cancer tissues with relatively high FOXO3a expression. Lower: immunostaining of cathepsin L was negative in gastric cancer tissues with relatively low FOXO3a expression. **b.** bivariate correlation analysis of the relationship between FOXO3a and cathepsin L expression level.

We detected the expression levels of FOXO3a and cathepsin L in 20 tissue microarray blocks containing 289 gastric cancer tissues (stage II/III) by immunohistochemistry (IHC). The results of IHC staining showed that 70.2% of the gastric cancer tissues with high expression of cathepsin L displayed high FOXO3a expression (Figure 4). Bivariate correlation analysis confirmed that the expression of cathepsin L was significantly positively correlated with FOXO3a expression in gastric cancer tissues. These data indicate that the upregulated cathepsin L is positively associated with FOXO3a overexpression in gastric cancer tissues, suggesting that FOXO3a may induce the transcription of cathepsin L to promote tumor invasion and metastasis.

### FOXO3a overexpression promotes gastric cancer cell migration and invasion via upregulation of cathepsin L

**Figure 5:**
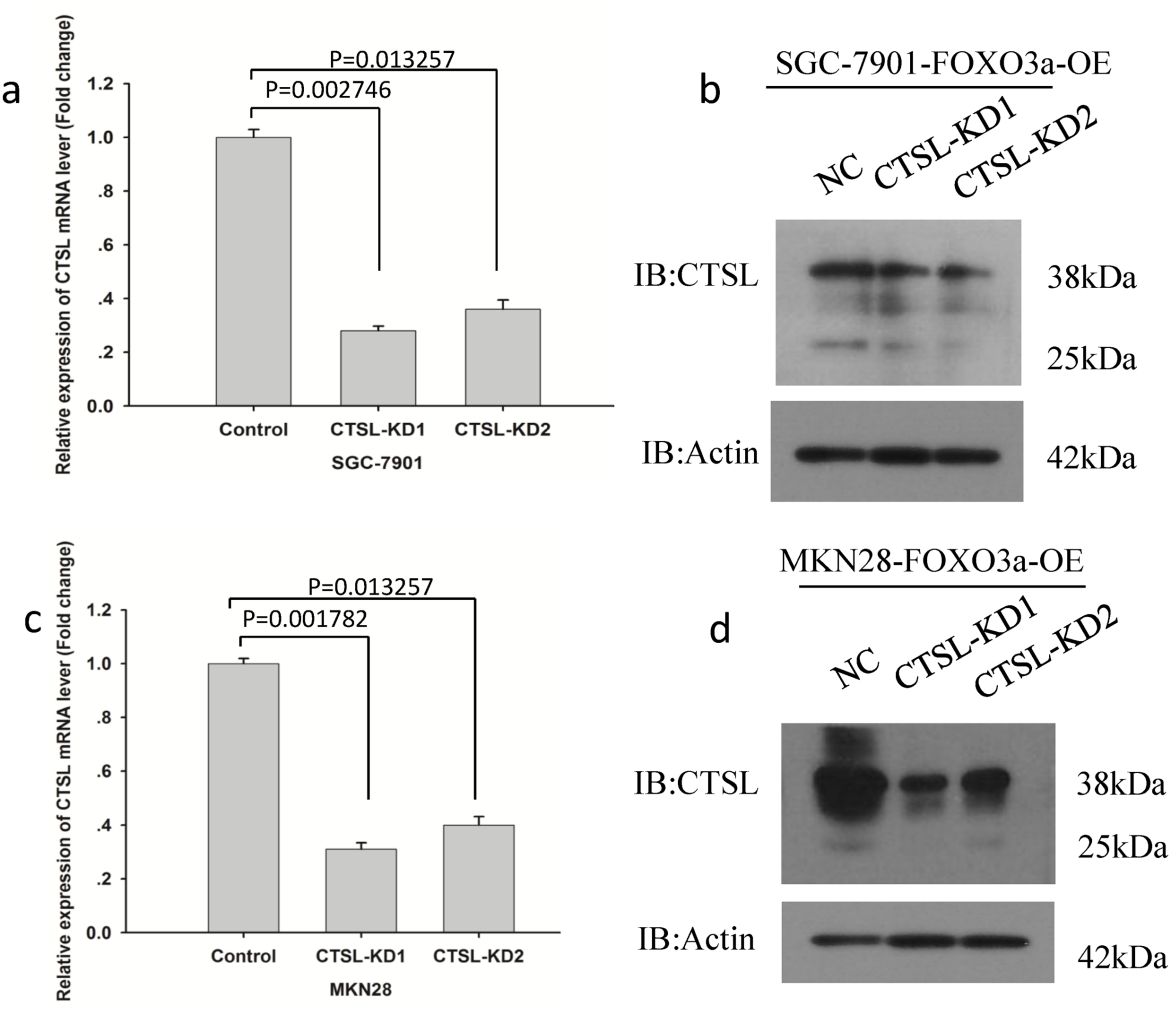
Knockdown of cathepsin L in gastric cancer cells **a-b.** Real-time PCR and western blot analysis of cathepsin L expression levels in SGC7901 cells with FOXO3a overexpressing and cathepsin L knockdown (KD) or negative control (NC). **c-d.** Real-time PCR and western blot analysis of cathepsin L expression levels in MKN28 cells with FOXO3a overexpressing and cathepsin L knockdown (KD) or negative control (NC).

**Figure 6:**
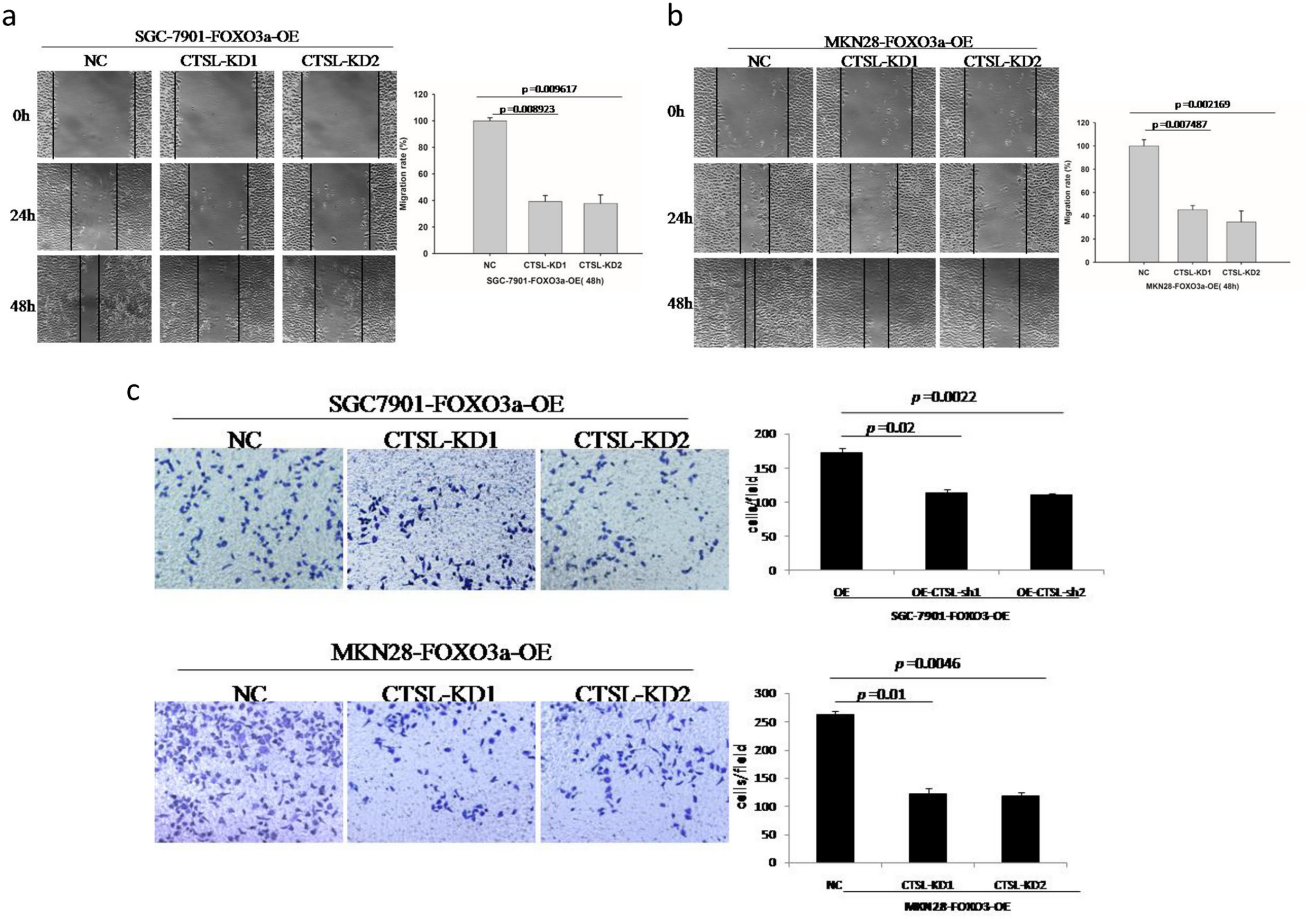
Migration and invasion assay of FOXO3a-OE and cathepsin L-KD cells The FOXO3a overexpressing cells with cathepsin L knocked down exhibited decreased cell migration **a, b.** and invasion **c.** compared to those FOXO3a overexpressed alone. Images are representative of three independent experiments. Values are mean ± s.e.m.

To determine whether upregulated expression of cathepsin L contributes to FOXO3a overexpression-promoted migration and invasion in gastric cancer cells, we transfected cathepsin L-specific knockdown constructs into FOXO3a-overexpressing SGC7901 and MKN28 cells (Figure 5). As shown in Figure 6, the FOXO3a-overexpressing cells with cathepsin L knocked down exhibited decreased cell migration and invasion compared with those of FOXO3a-overexpressing cells alone, indicating that cathepsin L can reverse the effect of FOXO3a on cell migration and invasion. Downregulation of cathepsin L did not have an effect on cell growth (Supplementary Figure 2).

### FOXO3a promotes tumor metastasis *in vivo*

**Figure 7:**
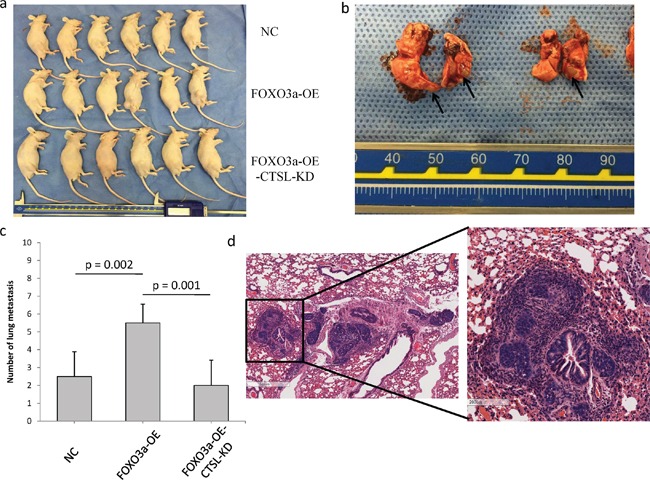
FOXO3a promoted gastric cancer cell metastasis *in vivo* **a.** SGC7901 cells (Nc, FOXO3a-OE, FOXO3a-OE-CTSL-KD) were injected in the lateral tail veins. There were six mice in each group. **b.** Lungs that were harvested from the mice that had been injected with FOXO3a-OE-CTSL-KD cells. The metastatic foci were labeled with arrows. **c.** The numbers of visible metastatic foci in lung of individual mouse. **d.** Representative hematoxylin and eosin staining of lung sections in FOXO3a-OE-CTSL-KD group.

To explore the effects of FOXO3a and cathepsin L on tumor metastasis *in vivo*, FOXO3a-OE SGC7901, FOXO3a-OE and cathepsin L KD SGC7901 and their corresponding control cells were injected into nude mice through the tail vein. FOXO3a overexpression significantly increased the number of visible metastatic foci in the lungs of each mouse (Figure 7). Silencing cathepsin L in FOXO3a-OE cells inhibited metastatic capability, as determined by the number of metastatic foci in the lungs of each mouse. Therefore, the *in vivo* results further confirmed that FOXO3a promotes gastric cancer metastasis by inducing cathepsin L expression.

### E-cadherin is downregulated by cathepsin L

**Figure 8:**
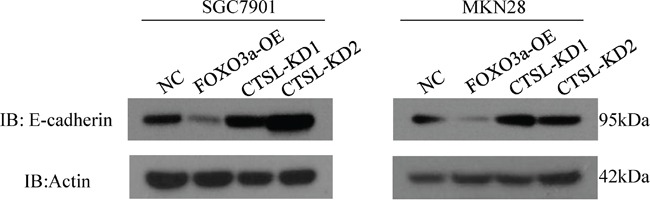
Western blot assay of E-cadherin in cathespin L knockdown or overexpression cells We have shown that cathepsin L expression is elevated in FOXO3a-OE cells, and E-cadherin expression is suppressed in these cells. In CTSL-KD cells, E-cadherin expression is elevated. This suggested a negative correlation between cathepsin L and E-cadherin in SGC7901 and MKN28 cells.

It has been reported that cathepsin L cleaves E-cadherin to reduce cell-cell adhesion and promote EMT in pancreatic cell carcinoma. To further demonstrate the molecular mechanisms by which cathepsin L promotes metastasis in gastric cancer, we analyzed E-cadherin expression in FOXO3a-OE and FOXO3a-OE-CTSL-KD cells by western blotting. As shown in Figure 8, cathepsin L was overexpressed in FOXO3a-OE cells which resulted in downregulation of E-cadherin. However, when cathepsin L was knockdown in FOXO3a-OE cells, expression of E-cadherin was increased, indicating that cathepsin L cleaves E-cadherin and participates in EMT of gastric cancer cells during the metastatic process.

### FOXO3a increases cathepsin L promoter activation

**Figure 9:**
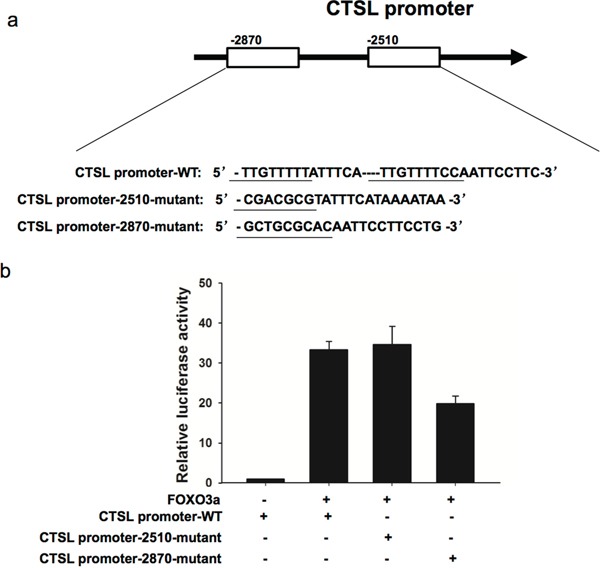
FOXO3a increased cathepsin L promoter activation pGL3-FOXO3a was transfected into MKN28 cells previously transfected with CTSL promoter-WT or CTSL promoter-2510-mutant or CTSL promoter-2870-mutant constract. Luciferase assays were performed to measure activity levels of three cathepsin L promoters. Three independent experiments were carried out to obtain standard deviation.

To confirm that cathepsin L is a transcriptional target of FOXO3a, we cloned three cathepsin L promoter fragments containing the wild type or mutant FOXO3a putative target site into pGL3-control vector. As shown in Figure 9, cotransfection of CTSL promoter-2870-mutant with pGL3-FOXO3a significantly reduced luciferase activity compared to that of CTSL promoter-WT with pGL3-FOXO3a, suggesting that FOXO3a bind on −2870 site of cathepsin L promoter.

## DISCUSSION

The major cause of mortality in patients with gastric cancer is metastasis, but the mechanisms underlying the multiple processes involved in metastasis remain unclear. Our study is the first to demonstrate the invasive function of FOXO3a in human gastric cancer. First, we found that expression of FOXO3a was significantly higher in gastric cancer tissues than in adjacent normal tissues [18]. Second, we conducted functional analyses to examine the FOXO3a function using stable FOXO3a-transfected cells. FOXO3a overexpression promoted cell migration and invasion in gastric cancer. These results suggest that FOXO3a may function as a tumor promoter and play an important role in cell motility in gastric cancer cells.

We also used luciferase assays to identify potential candidate targets of FOXO3a related to invasion and migration. Degradation of extracellular matrix (ECM) components is one of the most important processes in tumor invasion and metastasis. Malignant cells show increased proteolytic activity, which promotes digestion of the ECM. Cathepsins are a family of cysteine proteases. Once secreted into the extracellular space, cathepsins can degrade components of the ECM, allowing cancer cells to invade the surrounding tissues and blood and lymph vessels and metastasize to distant sites [28, 29]. We conducted luciferase assays and observed that luciferase activity was increased after cotransfection of FOXO3a and a 3′-UTR vector containing the cathepsin L FOXO3a target sequence. Cathepsin L protein expression was also positively correlated with FOXO3a and was significantly downregulated in FOXO3a-silenced SGC7901 and MKN28 cells, indicating that cathepsin L is a direct target of FOXO3a.

It has been reported that cathepsin L is expressed and secreted by gastric cancer and other types of tumors. Rousselet et al. found that antisense inhibition of cathepsin L completely abolished the invasiveness of human melanoma clones *in vitro* and inhibited metastasis *in vivo* [30]. By contrast, human glioblastoma clones transfected with cathepsin L constructs that displayed increased cathepsin L activity were significantly more invasive [31]. In addition to degrading the extracellular matrix directly, cathepsin L also reduces cell-cell adhesion through cleavage of E-cadherin [26]. In our study, we found that the expression of E-cadherin was correspondingly downregulated with increased levels of cathepsin L in FOXO3a-overexpressing gastric cancer cells, which is consistent with a previous report. Cathepsin L is essential for invasion and migration but not proliferation [26]. This result is consistent with our finding that cell migration in SGC7901 and MKN28 cathepsin L knockdown cells was significantly decreased compared with the control shRNA cells. However, cell growth in SGC7901 and MKN28 cathepsin L knockdown cells was not significantly different compared with the control shRNA cells.

Here, we demonstrated that FOXO3a promotes the migration and invasion of gastric cancer through induction of cathepsin L. However, recent publications have reported that increased FOXO3a expression may indicate good prognosis in patients with gastric cancer [18, 32]. The different conclusions may be due to the context-dependent roles of FOXO3a in the heterogeneous tissues of gastric carcinomas, which exhibit diverse biological properties at different stages. The inactivation of FOXO3a in the early stage of tumor growth by increased signaling through growth factors may offer a proliferative advantage to cancer. However, in later stages, stress conditions, such as serum deprivation, hypoxia, and oxidative stress, may reactivate FOXO3a and thus enhance tumor cell survival [19]. The nuclear β-catenin concentration is another important factor that may affect FOXO3a. Colon tumors with high nuclear β-catenin content can block the apoptosis-inducing role of FOXO3a and upregulate metastasis-related genes, including those involved in cytoskeleton remodeling, as well as cell shape and motility [20]. Unfortunately, in this study, we did not examine the expression of β-catenin in the nucleus of gastric cancer cells. We will conduct further studies to verify whether FOXO3a is regulated by other transcription factors, including β-catenin, in the process of gastric cancer metastasis.

Moreover, tumor therapy may also have an effect on the role of FOXO3a in malignant tumor development. Osuka S [33] found that FOXO3a promotes resistance to radiation during oncologic radiotherapy. Although FOXO3a is a good prognostic biomarker in patients receiving radical surgery, it may be a risk factor in patients with advanced gastric cancer, especially patients receiving radiation or chemotherapy. Future studies focusing on the effect of FOXO3a in advanced gastric cancer will be conducted.

In summary, our study unveiled a novel mechanism underlying the role of FOXO3a in promoting gastric cancer migration and invasion. We suggest that FOXO3a and cathepsin L may be potential therapeutic targets for blocking tumor metastasis.

## MATERIALS AND METHODS

### Cell lines and cell cultures

Gastric carcinoma cell lines SGC7901 and MKN28 were purchased from the Shanghai Cell Bank of the Chinese Academy of Sciences (Shanghai, China) and cultured in RPMI 1640 (HyClone, Logan, Utah, USA) supplemented with 10% FBS in a 5% CO_2_ humidified atmosphere at 37°C.

### Tissue array samples

A human gastric cancer tissue array was constructed as previously reported [18]. The expression levels of FOXO3a and cathepsin L were detected in gastric cancer tissues by histological and immunohistochemical analysis.

### Lentivirus-delivered shRNA gene knockdown

Lentivirus-delivered shRNA gene knockdown was performed as previously described [27]. Briefly, the shRNA sequences used for lentiviral silencing FOXO3a were 5′-GCTCTTGGTGGATCATCAA-3′ and 5′-CATGTTCAATGGGAGCTTGGA-3′. The cathepsin L silencing sequences were 5′-GGCGATGCACAACAGATTA-3′ and 5′-TGACACCGGCTTTGTGGAC-3′. The shRNA sequences were cloned into the pLenti lentiviral vector (Hanyin Co., Shanghai, China). The respective lentiviral vectors and packaging vectors were transfected into 293T cells for viral packaging. Sixty hours after transfection, the virus was collected to infect target cells in the presence of 8mg/ml polybrene (Sigma-Aldrich). Then, independent stable clones were selected and evaluated by western blotting.

### Retrovirus-mediated gene expression

FLAG-tagged FOXO3a was cloned into the pMSCV-IRES-GFP vector. For FOXO3a overexpression, cells were infected with viral supernatants from 293T cells transfected with FOXO3a or the control MSCV.

### Wound-healing assay

For wound-healing assays, cultured cells were resuspended and seeded in 6-well plates. A 200 μl pipette tip was used to make a scratch across the plate to form an artificial wound. Detached cells or cell debris were washed away after scratching. After 24, 48 and 72 hours, images of the wound areas under each condition were photographed. The percentage of wound closure was measured by the following formula (1-[current wound size/initial wound size])*100.

### Invasion assay

Invasion assays were performed using Transwell chambers with 8μm-pore size polycarbonate membrane filters. The upper surface of the filter was coated with Matrigel (Becton Dickinson, Bedford, MA). The Matrigel was dried at 37°C for 2 hours and placed at room temperature overnight. The cells were starved overnight, resuspended and seeded in the upper chamber at a density of 5 × 10^4^ cells/well. After 24 hours of transfection, the cells that invaded the lower surface of the filter were fixed and stained. Cells were counted in 10 randomly selected fields under a light microscope at high magnification. Experiments were repeated at least in triplicate.

### Western blotting

Proteins were extracted from log-phase cells and dissolved in 2 × SDS sample buffer. Proteins were quantified to ensure equal amounts were loaded. After electrophoresis, the proteins were separated and transferred to nitrocellulose membranes and blocked with nonfat milk. The membrane was incubated with the appropriate primary antibody (FOXO3a 1:1000 dilution, cathepsin L 1:1000 dilution) for 1 hour. The blot was washed three times with PBS, incubated with streptavidin-peroxidase for 15 min, and developed by the enhanced chemiluminescence method (Sigma, St. Louis, MO).

### Histological and immunohistochemical analysis

Twenty tissue microarray blocks were immunostained for FOXO3a and cathepsin L proteins and analyzed as previously described [18]. Briefly, immunohistochemistry was conducted using a highly sensitive streptavidin-biotin-peroxidase detection system with FOXO3a monoclonal antibody (1:100 dilution) and cathepsin L antibody (1:100 dilution). We quantitatively scored the tissue sections according to the percentage of positively stained cells as well as staining intensity.

### Luciferase reporter assay

Bioinformatics analysis was used to predict binding regions for FOXO3a on promoter of cathepsin L. Three segments of the promoter of cathepsin L (CTSL promoter-WT: 5′-TTGTTTTTATTTCA----TTGTTTTCCAATTCCTTC-3′, CTSL promoter-2510-mutant: 5′-CGACGCGTATTTCATAAAATAA-3′, CTSL promoter-2870-mutant: 5′-GCTGCGCACAA TTCCTTCCTG-3′) were cloned into pGL3-basic-Report (Promega, WI, USA) as well as human 3′-UTR of the FOXO3a gene. The reporter vectors containing the promoters of cathepsin L and pGL3-FOXO3a were transfected into MKN28 cells. After 48 h, luciferase activity was measured with a dual-luciferase reporter assay system (Promega, WI, USA).

### *In vivo* tumor metastasis assays

Sterile PBS (0.2 ml) containing approximately 1 × 10^6^ cells was injected into the tail veins of male nude mice. The mice were then monitored for overall health and body weight. Eight weeks after the injection, the mice were sacrificed, and their lungs were isolated. After counting the number of visible tumors on the lung surface, serial sections of the liver tissues were made and observed using imaging microscopy. There were 6 mice in each group.

### Statistical analyses

Correlation analyses were performed using the two-sided χ2 test or Fisher's exact test. Two-tailed Student's t tests were used to analyze differences between the protein overexpression and knockdown groups from *in vitro* experiments. A P-value <0.05 was considered statistically significant.

## SUPPLEMENTARY FIGURES


