# Supplementary material for: FOXO3a promotes gastric cancer cell migration and invasion through the induction of cathepsin L
Source: Oncotarget. 2016 Apr 25;7(23):34773–84. doi: 10.18632/oncotarget.8977 (PMC5085188; doi:10.18632/oncotarget.8977)
Supplement: Supplementary file 1 [file oncotarget-07-34773-s001.pdf]

SUPPLEMENTARY FIGURES

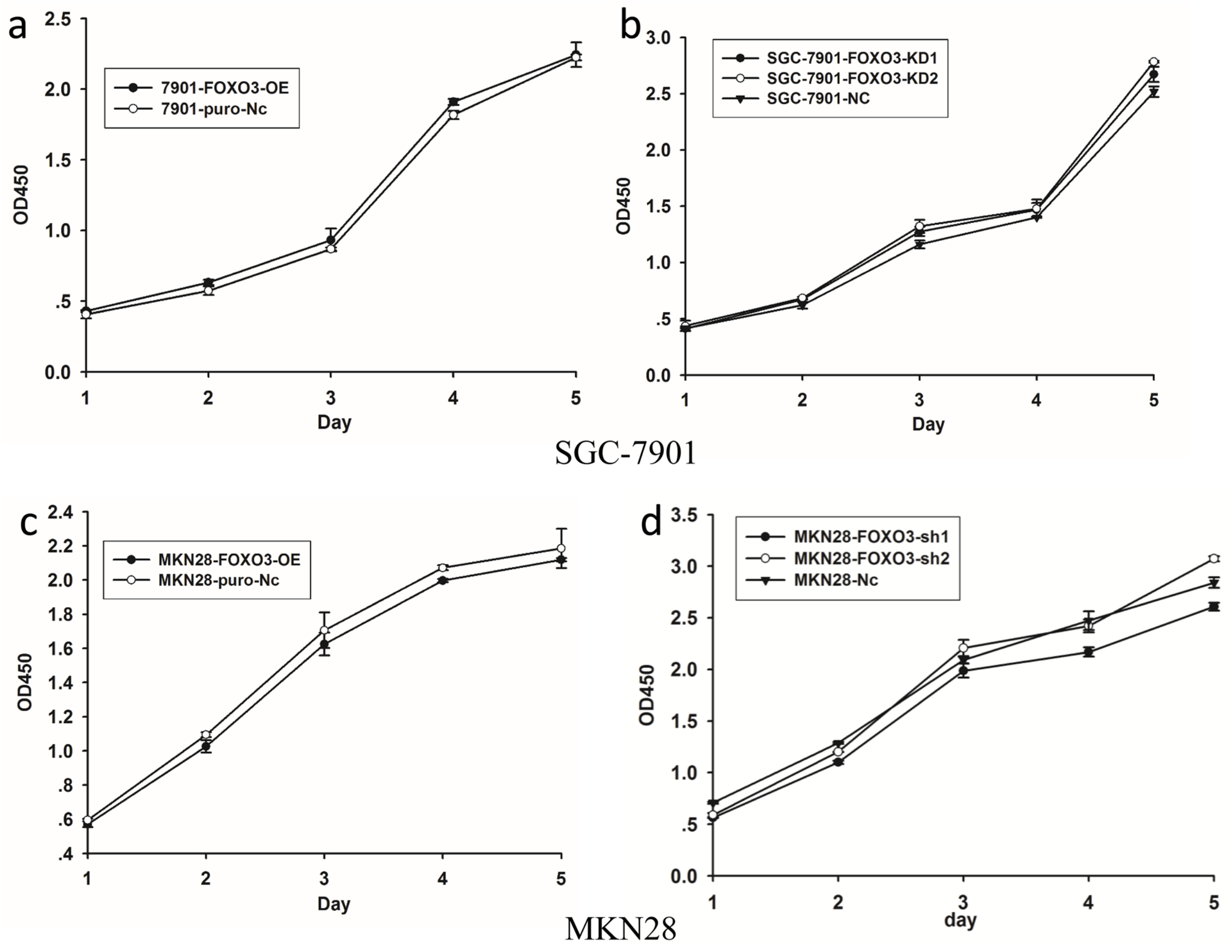

Supplementary Figure S1: FOXO3a has no effect on proliferation of gastric cancer cells. Cell growth of SGC7901 **a-b.** and MKN28 **c-d.** was measured using the MTT assay at various time points. Silencing or overexpressing FOXO3a has no effect on cell proliferation compared to negative controls.

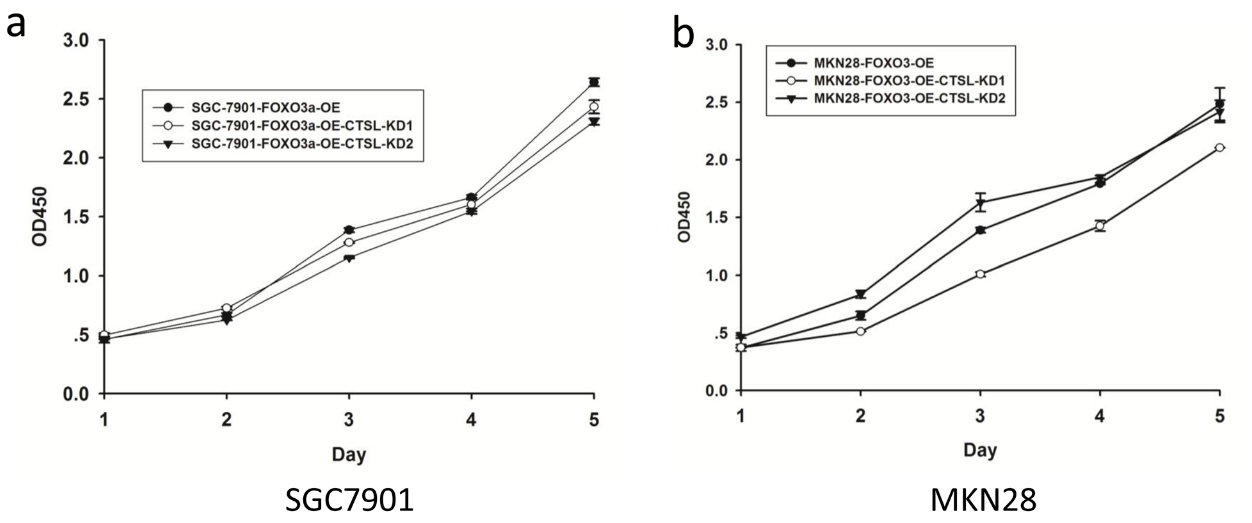

Supplementary Figure S2: Cathepsin L has no effect on proliferation of gastric cancer cells. Cell growth of SGC7901 **a.** and MKN28 **b.** in which FOXO3a was overexpressed was measured using the MTT assay at various time points. Silencing cathepsin L has no effect on cell proliferation compared to negative controls.
